# The Difficult Task of Diagnosing Depression in Elderly People with Cancer: A Systematic Review

**DOI:** 10.2174/1745017902117010295

**Published:** 2021-12-31

**Authors:** Elena Massa, Clelia Donisi, Nicole Liscia, Clelia Madeddu, Valentino Impera, Stefano Mariani, Mario Scartozzi, Eleonora Lai

**Affiliations:** 1 Department of Medical Sciences and Public Health, University of Cagliari, Cagliari, Italy

**Keywords:** Depression, Cancer, Elderly, Anticancer treatments, Quality of life, Suicide, Death risk

## Abstract

**Background:**

Depression is a common psychiatric problem in the elderly and oncology patients. In elderly people with cancer, depression has a peculiar phenomenology. It has a significant impact on the quality of life. Moreover, it is associated with poor adherence to treatments, increased risk of suicide, and mortality. Nevertheless, the topic of depression in elderly people with cancer remains unexplored.

**Objective:**

The main goal of this article is to review the literature from the past 20 years on the relationships between depression, cancer, and aging.

**Methods:**

The methods followed the Prisma model for eligibility of studies. The articles in which the keywords “depression”, “cancer”, “ elderly, aging, or geriatric” were present, either in the text or in the abstract, were selected. 8.056 articles, by matching the keywords “depression and elderly and cancer,” were identified. Only 532 papers met the eligibility criteria of search limits and selection process. Out of 532 papers, 467 were considered irrelevant, leaving 65 relevant studies. Out of 65 suitable studies, 39 (60.0%) met our quality criteria and were included.

**Results:**

The risk factors associated with depression in elderly people with cancer can be divided into 4 groups: 1) tumor-related; 2) anticancer treatment-related; 3) patients-related; 4) number and type of comorbidity. The main obstacles in diagnosing depression in elderly patients with cancer are the overlap of the symptoms of cancer and side effects of treatment with the symptoms of depression but also the different ways of reporting depressive symptoms of elderly people and the different clinical types of depression. There is a lack of data regarding validated scales to assess depression in geriatric patients with cancer. Any mental illness, specifically co-occurring anxiety and depression, increases the risk of diagnosis delay and anticancer treatment adherence. Cancer and the diagnosis of mental disorders prior to cancer diagnosis correlate with an increased risk for suicide. A non-pharmacological therapeutic approach, pharmacological treatment and/or a combination of both can be used to treat elderly patients with cancer, but a detailed analysis of comorbidities and the assessment of polypharmacy is mandatory in order to avoid potential side-effects and interactions between antidepressants and the other drugs taken by the patients.

**Conclusion:**

Future research should be conducted with the aim of developing a modified and adapted assessment method for the diagnosis and treatment of depression in elderly people with cancer in order to improve their clinical outcomes and quality of life.

## 1 INTRODUCTION

Advanced age is a significant risk factor for cancer. Epidemiologic studies describe that about 60% of all malignancies occur in people aged 65 years or older [1-3]. The proportion and number of geriatric cancer patients are expected to increase significantly (by 67%) over the next 20 years [4]. Cancer and related symptoms, its treatment, and side effects can have a significant negative impact on the physical functioning and mental health of patients. For the elderly, this impact may be further exacerbated by the presence of other medical problems and the use of multiple medications for their control, thus dramatically affecting their quality of life during the cancer experience [5].

Depression is a condition characterized by the presence of low mood or loss of interest and is the most common cause of emotional distress both in the geriatric population and in oncology [6-13]. Depression is associated with decreased quality of life, significant deterioration of recreational and physical activities, relationship difficulties, and sleep disorders [14]. Besides, in people with cancer, it is also associated with reduced adherence to anti-cancer treatment and prolonged length of hospital stay [15-20].

In several studies, depression was found to be an independent predictor of poor survival in patients with advanced cancer [21-23], and a strong association between MDD (Major Depressive Disorders) and early death in people with cancer is reported by Sancassiani *et al*. [24]. On the other hand, the suicide risk in cancer patients seems to be higher than in patients with other medical illnesses [25-40].

Despite the high number of published articles, the exact prevalence of depression in elderly people with cancer remains unclear and difficult to estimate [41-53]. Different studies reported ranges from 3-31% [54-57]. This wide range of depression is the consequence of the following methodologic differences reported by the published studies: 1) different diagnostic criteria for depression, 2) different measurement strategies, 3) different cancer types and disease stages of the sample studied, 4) different ages of the patients, 5) the lack of clarity on whether the depressive symptoms were already present or it was a new clinical diagnosis, 6) the lack of coherence in the timeline of the prevalence assessment in the course of disease (diagnosis, before or after antineoplastic therapy) [58, 59]. The main goal of this article is to review the literature from the past 20 years on the relationships between depression, cancer, and aging. After an overview of the risk factors associated with depression in elderly people with cancer, we focused on the detailed analysis of the following aspects: difficulties in recognition of depressive symptoms in elderly people with cancer, the tools used for the evaluation of depression, the impact of depression on adherence to antineoplastic treatments and also on the outcome and prognosis of elderly people with cancer and finally, the suicide risk in depressed elderly people with cancer.

## 2 METHODS

### 2.1 Eligibility Criteria

The methodology of this study follows the PRISMA model (preferred reporting items for systematic reviews and meta-analysis) for the determination of eligibility [60].

- Inclusion criteria and type of studies were: subjects aged 65 and older; clinical trials, meta-analysis, randomized controlled trials, reviews, and systematic reviews with a specific focus on depression in elderly people with cancer. We selected only articles written in English, published between 2000-2020, and for which we could find the full paper.

Exclusion criteria: 1) articles not written in English; 2) articles with non-cancer population; 3) articles focused on other medical symptoms (*e.g*., pain, sexual dysfunction, anorexia, malnutrition, *etc*.), other conditions that can be associated with depression (*e.g*., cognitive impairment, symptom beliefs, coping resources), social factors (*e.g*., social support); 4) articles with a primary focus on outcomes (quality of life); 5) articles with a primary focus on survivorship; 6) articles with children population and patients <65 years of age.

### 2.2 Sources of Information

The articles included in this study were selected on searches in the database PubMed in January 2021. Additionally, the reference lists of all the studies were manually searched and selected for inclusion in the review.

### 2.3 Search

For our purpose, we used the following search terms: “cancer”, “elderly”, “aging”, “geriatric”, depression”, “chemotherapy”. We selected only the articles in which the keywords “depression”, “cancer”, “ elderly, aging or geriatric” were all present either in the text or in the abstract. All papers selected during the search procedures were collected into a database, and their titles and abstracts were screened to determine if each of them met the selection criteria. This process was conducted by two researchers independently. Moreover, the decision to include each article was approved by both researchers. Conceptually related articles were also included. A paper was considered “conceptually related” to the issue of depression in elderly people with cancer if it provided information (data, models, hypotheses) on a topic that has been reported in the literature to be related to the abovementioned keywords. We identified 83.890 articles by matching the keywords “depression and elderly”, 7652 articles by matching the keywords “depression and geriatric”, 11.317 articles by matching the keywords “depression and aging”, 481 articles by matching the keywords “depression and geriatric and cancer”, 678 articles by matching the keywords “depression and aging and cancer”, 8.056 articles by matching the keywords “depression and elderly and cancer”, 2617 by matching the keywords “depression and elderly and cancer and chemotherapy”.

Among the 8056 articles selected, only 532 papers were eligible to be reviewed because they met the above-mentioned criteria of search limits and selection process. After fully reviewing the articles, 467 were considered not relevant (the most common reason was that the selected papers were not strictly connected with the topic of depression in elderly people with cancer), leaving 65 relevant studies. Out of 65 suitable studies, 39 (60.0%) met our quality criteria and were included Prisma Flow Diagram is reported in Fig. (**1**).

### 2.4 Characteristics of Samples in the Studies

We focused the report on 39 articles as shown in the PRIMA diagram [60]; 17 of the studies were reviews, 15 clinical trials, 2 meta-analysis, 1 cohort study, 2 retrospective analysis, and 1 comparative study. The topics covered by the 39 studies were divided into 6 groups: risk factors for depression in elderly people with cancer, the main obstacles in diagnosing depression, the lack of validated tools to detect depression, the impact of depression on adherence to anticancer treatments, risk of suicide, therapeutic approaches available for treating depression in this special group of patients.

### 2.5 Search of Data

The following data were extracted from the articles: sample size, characteristics of participants, type of intervention, and meaningful main results. In addition to these, several other information about methods and results were collected. These procedures were performed by two independent investigators, who reached a consensus in the case of divergence.

## 3 RESULTS

### 3.1 Risk Factors for Depression in Elderly People with Cancer

**S**everal studies tried to identify associated risk factors for depression in elderly people with cancer, with considerable difficulties due to the frequent overlapping of symptoms attributable to both depression and cancer [3, 5, 19, 46, 47, 54, 56, 61-67]. A history of previous depression and depression treatment, family history of depression and suicide, female gender, social isolation, the loss of a spouse, friend or relative, low educational levels, dependency, cognitive disorders, and vascular risk factors have been described as potentially associated with depression in elderly people with cancer. A higher risk of depression has been reported in older people with pancreatic, head and neck, lung, and gynecological malignancies than with other primary tumors, as well as in those with higher severity of cancer-related symptoms, lower levels of prior physical functioning, and increased physical impairment or discomfort [5, 13, 50, 62, 63, 65, 66]. Additionally, some anticancer drugs have been associated with depression: corticosteroids (as premedication or as an active part of treatment in hematological malignancies), vinca alkaloids (vincristine and vinblastine), L-asparaginase, procarbazine, cyproterone acetate, endocrine therapy like tamoxifen [63, 66]. However, studies differ widely in the inclusion criteria and in the methods of depressive status assessment, thus complicating the possibility to draw definitive conclusions and data [19].

Moreover, data about the relationship between age and risk of depression in older people with cancer are not univocal [66]. A bidirectional mechanism of interaction between aging, cancer, and depression, which involves primarily neuro-immune-endocrine system dysregulation, was discussed by Spoletini *et al*. Aging is associated with sympathetic/ parasympathetic deregulation, hypothalamic-pituitary-adrenal (HPA) axis activation by stress conditions, immune deregulation leading to lymphocytes T helper1/2 imbalance, up-regulation of pro-inflammatory cytokines (IL-1, TNF-α, IL-6, IL-8, and IL-18), down-regulation of anti-inflammatory cytokines (TGF-β, IL-10), and modifications of natural killer cells activity [3]. More particularly, dysregulation of the thyroid axis, changes in neurotransmitter activity and metabolism, desynchronization of circadian rhythms, and physical/cognitive decline are common with aging, as well as comorbidities (such as congestive heart failure, neurosyphilis, hypercalcemia, hyperthyroidism, Cushing disease) and consequently polypharmacy [63].

Recently, more and more studies suggest that dysregulated thyroid function may be involved in the pathogenesis of anxiety and depression, such as hypothyroidism is reported to correlate with increased anxiety and depression severity, and the TSH level is elevated in patients with anxiety/depression compared to healthy subjects. These data all indicate that thyroid function or thyroid hormone may play a critical role in the development of anxiety and depression. A possible role of thyroid autoimmunity in the pathogenesis of depression has been hypothesized. A prevalence of up to 20% of elevated titers of antithyroid antibodies has been documented in depressed patients in several reports compared to a 5–10% prevalence in the general population. However, this should be viewed with caution since these reports either lacked a control group or showed no significant difference between the group with an affective disorder and the control group with a nonaffective psychiatric disorder [64].

As reported by Nelson N.J. *et al*. in 736 older prostate cancer patients, depressive symptomatology significantly increased with age, which was confirmed as a significant predictor of anxiety and depression after controlling for multiple variables, including functional well-being [66]. A cross-sectional study by Goldzweig *et al*. investigated the association between age and frequency of depression in this category of patients and the influence of other variables on this association. Globally, 243 patients were enrolled. The multivariate binary logistic regression for predicting depression and distress incidence showed that the only significant predictors for depression and distress were functional status, social support, and age group. More specifically, belonging to the group of patients ≥ 86 years was a significant predictor factor for depression compared to younger patients [67]. Presumably, subjects’ self-perception of age might play a key role, considering that age perception was shown to be related to mortality [68]. Another study evaluated the association between age, anxiety, and depression in 500 older cancer patients. In the univariate analysis, depression was correlated to social support (r = −0.28, p < 0.0001), number of comorbidities (r = 0.27, p < 0.0001) and disease stage (M = 3.1 early-stage *vs*. 3.9 advanced stage, p = 0.005). The correlation among the same factors and depression was confirmed by the multivariate analysis (social support (β = −0.04, p < 0.001), number of comorbidities (β = 0.50, p < 0.001), and advanced stage (β = 0.83, p = 0.003). Conversely, age was not associated with depression in either analysis, as if advanced disease (61.4% stage IV) and multiple medical comorbidities (44.2% of patients had ≥ 3 conditions, 68.4% had ≥ 2, and 31.6% had < 2) eclipsed age as a predictor of depression in these patients [56].

A systematic review by Parpa *et al*. showed that depression was most common among patients with advanced cancer stage, uncontrollable pain, and poor physical status [45, 69, 70]. In the prospective study by Atag *et al*., 170 cancer patients on active chemotherapy with a mean age of 71 years were evaluated through the Yesavage Geriatric Depression Scale to examine the prevalence of depressive symptoms and to determine the possible associated factors. A high depressive symptom score was reported in 19.4% of patients. When comparing the group with low (group 1) and high depressive symptom scores (group 2), the only related variable among those with a high depressive symptom score was pain. The mean pain score was 3.15 ± 2.77 in group 1 and 1.81 ±1.88 in group 2 (p = 0.012). The presence of congestive heart failure, although showing high depressive symptom prevalence (30.3%), did not reach statistical significance (p = 0.078) (8). The presence of pain has been demonstrated to increase the prevalence of depression in cancer patients, and depressed patients are, in turn, more sensitive to cancer pain [3, 71].

The systematic review of Waguih *et al*. [72] reported that patients with pain and depression experience reduced physical, mental, and social functioning as opposed to patients with only depression or only pain. While the precise biological mechanisms between pain and depression are still being investigated, there are indicative trends and symptomatic relationships. Since pain induces physical and psychological distress, the causative-relationship hypothesis between depression and pain is supported. There might be an underlying neurobiological relationship between pain and depression. It is worth noting, however, that physical pain alone may not be the only causative factor, and that psychological “pain” could also further exacerbate the conditions of a patient with depression and, in turn, cause physical pain. Kurtz *et al*. conducted a study on 420 patients aged between 65 and 98 who had received an incidental diagnosis of breast, colon, lung, or prostate cancer, with the aim of identifying which elderly people with cancer were more likely to develop problems with physical and physiological functioning in association with the tumor itself and its treatment. The covariate analysis showed that the only significant factors predicting depression were cancer symptom severity and pre-diagnosis physical functioning; more specifically, patients with high levels of symptom severity and low levels of pre-diagnosis physical functioning had an increased risk of depression. Conversely, primary tumor site, gender, disease stage, and treatment type were not significantly related to depression development in the study population [5].

Duc *et al*. conducted a prospective, multicenter cohort study in elderly people with cancer (70 years and older) who were receiving first-line chemotherapy with the aim to identify socio-demographic, clinical, and treatment-related factors of depression in the elderly during chemotherapy in order to detect early and treat these patients consequently. Moreover, the authors assessed the correlation between the efficacy and safety of chemotherapy with depression. Depressive symptoms were measured by the Geriatric depression scale (GDS) -15; patients were also evaluated with Comprehensive Geriatric Assessment (CGA) including, Mini-mental State Examination (MMSE), Mini Nutritional Assessment (MNA), activities of daily living (ADL), instrumental activities of daily living (IADL), and timed get up and go (TUG) at baseline before the treatment and before the fourth chemotherapy cycle. Globally, 364 patients were included in the study, with a median age of 77.6 years. 46.2% of patients had depressive symptoms at baseline, of which 23.0% were no longer depressed during chemotherapy; conversely, 44.6% (of whom 25% were not depressed at baseline) showed depressive symptoms after 4 cycles; 42.7% did not have depressive symptoms at all. At univariate analysis, older age (p = 0.004), living alone (p = 0.009), being unmarried (p = 0.005), female sex (p < 0.001), lymphomas, colon or pancreatic cancer (p = 0.007), ineffective chemotherapy, high GDS-15 at baseline, low MMSE, low MNA, low ADL, and slow TUG (p ≤ 0.001) were significantly associated with a higher risk of depression; these variables were selected for the multivariate analysis. In the final multiple models adjusted for age and sex, independently associated factors with depression were: GDS at baseline, MNA, living alone, and the effects of chemotherapy; in the complete case analysis (195 patients), the presence of baseline depressive symptoms (OR= 6.4, p < 0.001), baseline poor nutritional status (OR= 6.2, p = 0.03), and living alone (OR= 2.31, p = 0.03) were correlated with an increased risk of developing depression during chemotherapy. Conversely, a non-statistically significant trend was observed between effective chemotherapy (*i.e*., partial or complete response) and a lower risk of depressive symptoms (OR= 0.48, p = 0.054). After multiple imputation, estimated parameters were similar for GDS-15 (OR= 6.93, p < 0.001) and MNA at baseline (OR= 5.54, p = 0.02), whereas living alone was no longer significantly associated with development of depression during treatment (OR= 1.44, p = 0.262), and statistical significance was reached for effective chemotherapy with a lower risk (OR= 0.42, p = 0.018) [47].

### 3.2 Obstacles in Diagnosing Depression in Elderly Patients with Cancer

Identifying, assessing, and treating depression in elderly people with cancer is difficult and presents a challenging task for clinicians because it combines the complexities of diagnosing depression in older adults with those of detecting depression in people with cancer [57]. There are several barriers to the identification and diagnosis of depression among older adults in medical settings [73]. Firstly, somatic symptoms and depressive cognitions are often viewed as a normal part of the aging process in older adults. Secondly, cognitive dysfunction can obscure depressive symptoms, and lastly, older adults may report depressive symptoms differently than younger adults [7, 74, 75]. Depression in the geriatric population with cancer may have a peculiar phenomenology; the moderate rate of major depressive disorder (MDD) and the high rate of minor depressive disorder are accompanied by subthreshold forms of depression that are at risk of being under-recognized and untreated [76, 77]. Two different studies reported that between 15 and 36% of community-dwelling older adults endorsed subthreshold (*i.e*., not meet the threshold for MDD) depressive symptoms [6, 78]. These less severe forms of MDD (*i.e*., minor, subsyndromic or subthreshold depression, dysthymia, or adjustment disorder with depressed mood) can create a significant impairment and decrease in quality of life as they are often associated with greater disability, morbidity, and mortality among the elderly [74, 75, 77]. Additionally, older adults with subthreshold depressive disorders report impairments in physical, social, and role functioning that are similar to MDD [78].

Variants of traditional MDD have been reported in individuals in whom depression is first diagnosed at the age of 60 and older (late life depression - LLD). Gallo *et* *al*. characterized depression in older adults as “depression without sadness”, noting that depressed older adults manifest irritability or withdrawal more often than dysphoric mood [79, 80]. These authors described non-dysphoric depression as a syndrome that includes apathy, anhedonia, fatigue, sleep disturbance, and other somatic symptoms. The authors concluded that non-dysphoric depression is as likely as classic MDD to generate significant distress and impairment in older adults.

When compared with younger patients, elderly patients often present specific features of depression with more somatic symptoms of depression (*i.e*., sleep problems and stomach ache) as opposed to affective complaints such as self-criticism, guilt, sense of failure, and sadness [3, 6, 12, 13, 80]. Consistent with these data, a meta-analysis by Hegeman *et al*. found that, compared to young adults with depression, older adults with depression tend to experience more psychomotor agitation, hypochondria, gastrointestinal somatic symptoms, and general somatic symptoms [81].

In patients with cancer, the main difficulty in diagnosing depression is that many symptoms of cancer and side effects of treatment overlap with the symptoms of depression [82]. The diagnostic and statistical manual of mental disorders - DSM-5 criteria [83] used to define depression include several somatic items such as significant weight loss, sleep problems, fatigue/anergia, difficulty with concentration, and thoughts of suicide (Table **1**). The same symptoms may be either symptoms of cancer and/or treatment side effects or from some combination of these three.

In order to help distinguish between the somatic symptoms of depression and the side effects of the disease, it may also be helpful to discuss symptoms suggested by Guo *et al*. [83, 84], such as late insomnia, mood variation, anxiety, and loss of sexual interest, which offer succinct and specific evidence for a diagnosis of depression in cancer patients.

Depression in elderly patients also correlates with social disconnection, which includes both social withdrawal and loneliness. Older adults who experience social isolation are at greater risk of increased morbidity, decreased immune function, depression, and cognitive decline [85, 86]. Loneliness has been particularly closely linked to depressed mood, decreased well-being, and even higher mortality.

Patients may not immediately recognize any of these symptoms as being related to depression. Thus, clinicians must carefully consider these symptom relationships when evaluating their older patients [75, 79, 83, 87].

Considering these difficulties, it is not surprising that late-life depression is underdiagnosed and underrecognized. Even when diagnosed, under-treatment of psychological distress among older people is also common, as reported by Ellis J *et al*. in 326 patients with metastatic GI or lung cancer. Older age was found to be associated with a lower likelihood of being referred to specialized psychosocial cancer care regardless of the level of distress. Only 22% of patients aged ≥ 70 were referred compared with 100% of patients aged ≤ 40 years [87].

### 3.3 Tools (Instruments) To Assess Depression in Elderly People with Cancer

In order to screen cancer patients for depression, Weidenberg *et al*. suggest asking the two gateway questions of depressed mood and loss of interest or pleasure. These are defined as the two “pure” symptoms of depression in cancer patients [57]. Mitchell *et al*. examined data from 17 studies with the aim of defining the validity of using one or both of these questions in detecting depression in cancer settings. The findings showed that the use of both questions assessing, respectively, “sad mood” and “loss of interest” had a sensitivity of 91% and a specificity of 86%, with a positive predictive value of 57% and a negative predictive value of 98% [88]. Older depressed patients are less likely to endorse the two gateway symptoms of depressed mood and loss of pleasure or interest. Several authors have contended that DSM-5 criteria (Table **1**) used for the depression of diagnosis may underestimate depression in older adults based on its emphasis on depressed mood as a gateway symptom [75, 79, 83].

Useful information on the presence and characteristics of depression in elderly patients with cancer can be obtained from the Comprehensive geriatric assessment (CGA), a method of assessment used by geriatricians and oncologists to describe the multidisciplinary assessment of an elderly patient. CGA is recommended as a part of the standard assessment of an older adult considering chemotherapy or for assessing an elderly patient's suitability for a clinical trial, given that it is more thorough and tailored to the needs and problems of older patients than is KPS [89]. There are four well-validated, self-report scales (measures) that are commonly used to assess depression: Geriatric Depression Scale [90], the Hospital Anxiety and Depression Scale (HADS) [91], the Center for Epidemiologic Studies on Depression CESD-20 [92], the Beck Depression Inventory [81].

The Geriatric Depression Scale [90] is a multi-item scale with high sensitivity and specificity in the elderly community and primary-care samples, specifically designed for use with aged populations. Furthermore, it uses simple and consistent response alternatives and focuses on non-somatic symptoms in order to minimize overdiagnosis in medically ill populations [71]. The majority of other multi-item scales used in palliative care do not share these items. All these validated scales are easy and quick to administer and will likely provide a clinician with a baseline measure of depressive symptoms. However, as reported by several authors, these screening measures are inadequate for identifying depression in a geriatric cancer population, and no clear data are available regarding which scale is the most appropriate to assess depression in geriatric patients with cancer [93]. Further validation and measure development with older cancer patient samples are needed. Therefore researchers and clinicians should use caution when selecting depression measures for geriatric cancer patients, as the risk of underestimating depression is substantial [94].

### 3.4 Effect of Depression on Cancer Diagnosis and Treatment in Elderly People with Cancer

Despite the majority of cancer diagnoses and morbidity occurring in patients aged 65 years and older [95], elderly patients remain underrepresented in cancer clinical trials and clinically untreated for cancer [96-99]. The correlation between anxiety and depression and noncompliance with treatment recommendations of medical patients is well documented [100]. However, the data discussing the possible role of depression on delayed diagnosis and cancer treatment, as well as its possible role in non-compliance with medical treatments, are very limited in cancer patients, especially in elderly people with cancer.

Iglay K *et al*. carried out a study with the aim of comparing diagnosis and treatment delays in elderly women with breast cancer with and without pre-existing mental illness. The study suggests that patients with any mental illness face an increased risk of adjuvant chemotherapy delay compared to patients without a mental illness. Although insignificant, a trend for this association was seen for each category of mental illnesses that were examined, except for depression. An increased risk of diagnosis delay was observed for co-occurring anxiety and depression, while an increased risk was not seen for patients with anxiety alone or depression alone, suggesting the presence of a synergistic effect concerning these conditions. Depression and anxiety are both associated with a perceived low level of self-efficacy [101]. Consistent with these results, Burgess *et al*. interviewed 158 patients with breast cancer and showed that the presence of depression or anxiety alone did not delay presentation to the physician after breast cancer symptom discovery [102]. In contrast, the result of a retrospective study by Goodwin *et al*. showed that a prior diagnosis of depression was not associated with delays in diagnosis of breast cancer, but women with a prior diagnosis of depression were less likely to receive treatments that are generally recognized as appropriate and also had poorer survival than women without a diagnosis of depression. Decreased survival associated with depression remains after controlling for other factors that might influence survival, but it is not explained by the greater risk of receiving less-than-definitive treatment in women with a prior diagnosis of depression. Finally, women with a recent diagnosis of depression are at greater risk of receiving non-definitive treatment and experiencing worse survival after a diagnosis of breast cancer. Nevertheless, the differences in treatment do not explain worse survival [103].

### 3.5 Suicide in Elderly People with Cancer

In a retrospective study concerning suicide in elderly patients with prostate cancer, Lorente *et al*. [104] found that 20% of all suicides were due to cancer and that elderly men with prostate cancer were at increased risk for committing suicide, especially those with depression and/or anxiety symptoms and uncontrollable pain. In Miller *et al*.’s study [105] regarding suicidal risk in Americans aged 65 while controlling for medical and psychiatric comorbidity, cancer was the only medical factor associated with suicide. In addition, a suicide rate of 23% was present among patients with prostate cancer [106]. In a retrospective cohort study, Choi JW *et al*. [107] investigated suicide risk within 1 year after cancer diagnosis in older adults. In the total sample of 259,688 older adults, 36.1% of total suicide deaths occurred within one year after cancer diagnosis; 64,922 older patients with cancer showed higher suicide risk after cancer diagnosis compared to non-cancer patients. Patients with mental disorders before being diagnosed with cancer were at more increased suicide risk than cancer patients without mental disorder diagnosis compared to non-cancer participants. Suicide risk among older patients with bladder, head and neck, liver, lung, and stomach cancers was higher than in non-cancer participants.

As reported by Labisi *et al*., elderly patients with advanced cancer should be monitored for suicide if risk factors are present and particular attention should be paid to the removal of risk factors for suicide. A prerequisite for developing a treatment plan aimed at managing the patient exhibiting symptoms of suicidality is to conduct an assessment that provides information about the patient’s level of risk to harm or kill him/herself. Predicting suicide is difficult, but the following are identified risk factors that have been linked to increased suicide risk: (a) age (*i.e*., elderly and adolescent); (b) alcohol dependence; (c) history of suicide attempts, especially those requiring life-saving medical intervention; (d) a diagnosed psychiatric disorder combined with social isolation or a recent loss of an intimate relationship [66]

### 3.6 Treatment of Depression in Elderly People with Cancer

The management of depression in elderly patients with cancer is mainly based on data derived from the general population since, unfortunately, no specific guidelines for this set of patients are available yet, and randomized clinical trials are lacking [3, 19]. Nevertheless, sufficient evidence is available to identify an adapted therapeutic approach, and these patients should be referred to appropriate mental health services.

In elderly patients with cancer, depression can be treated with a non-pharmacological therapeutic approach, a pharmacological treatment and/or a combination of both, according to the baseline assessment of depression and its evolution [3, 19, 63].

Non-pharmacological intervention involves psychotherapy. It aims to help patients to adapt to stressful situations and to improve their coping abilities [107]. Psychotherapy includes supportive psychotherapy, cognitive behavior therapy (CBT), and problem-solving therapy (PST). They are generally recommended in older cancer subjects, which are believed to benefit from structured exercises, skill development, and behavioral activation [19, 107].

Supportive techniques might already be performed by oncologists and oncology nurses, with active listening and supportive comments [108]. CBT explores the way patients develop depression, starting from maladaptive patterns of thinking and assessment of their status. It has the goal of restructuring dysfunctional thoughts and helping patients build an adapted perspective of their situation. CBT also comprises relaxation techniques [19, 69, 109]. PST aims at improving patients’ problem-solving skills in order to develop a better coping mechanism [19].

Group therapy involving not only elderly people with cancer but also their caregivers and families might be helpful, too, leading to reciprocal support from other people facing the same or similar conditions. Larger studies are needed to demonstrate the role of group therapy in improving the survival rate, as evident from some observations [19, 109].

Psychotherapy should be adapted to patients’ ability to attend the sessions, to their clinical conditions and cancer-related symptoms, as well as cancer treatment schedules. Moreover, the different modalities should be chosen based on patients’ characteristics and logistic/human resources available. Furthermore, testing in larger groups of elderly people with cancer should be specifically explored [19]. If patients do not benefit from these techniques, they should be referred to a social worker, a psychologist, or a psychiatrist according to the gravity of depression symptoms. An integrated pharmacologic approach should be considered as well.

Globally, in case of very high levels of distress, inability to perform daily activities, and a failure of psychotherapy, the introduction of a pharmacologic treatment should be considered. The decision of starting an antidepressant drug and the choice of the most appropriate agent in elderly patients with cancer is based on several factors, such as previous response to an antidepressant in the past, patients’ global state of health and cognitive abilities, social and financial conditions, and other psychiatric disorders (anxiety, substance abuse, psychosis). The analysis of comorbidities and the assessment of polypharmacy are crucial in order to evaluate potential life-threatening side-effects and interactions between antidepressants and the other drugs taken by the patients [70, 109].

The antidepressant administration in the specific population of depressed elderly patients with cancer is still under study. However, some suggestions about how these drugs should be administered in this patients’ setting can be drawn from data available for elderly subjects and for cancer patients [62]. The first choice should be selective serotonin reuptake inhibitors (SSRIs). SSRIs (citalopram, fluoxetine, paroxetine, sertraline) have a more favorable safety profile than other antidepressants, thus leading to better compliance. Moreover, they can also be administered in case of ischemic heart disease, prostatic hypertrophy, uncontrolled glaucoma, which can be common in older age. As a second choice, venlafaxine, mirtazapine, and bupropion can be considered, whereas nortriptyline and desipramine should be reserved as a third option in case of severe melancholic depression.

Conversely, other tricyclic antidepressants, monamine oxidase inhibitors (MAOIs), should be avoided in this patients’ setting. In fact, tricyclic drugs might lead to cardiac arrhythmias, urinary retention, and constipation due to the anticholinergic effects; as for MAOIs, they require dietary restrictions in order to avoid tyramine excess, which may provoke a hypertensive crisis. Moreover, MAOIs may provoke myoclonus and delirium when combined with opioid analgesics, which are often administered for cancer pain treatment [64, 109].

The rule for dosing antidepressants in elderly people with cancer is “start low and go slow”. Patients have to be instructed that it takes between 2 to 6 weeks to have the anti-depressant effect, and then the dose may be titrated if tolerated and if necessary. This obviously requires strict monitoring of patients’ symptoms and conditions. Only this way the occurrence of poor compliance can be avoided or at least reduced [19, 110]. Along with poor compliance, other barriers to the treatment of depression in elderly cancer patients may be related to polypharmacy, difficult patient/doctor relationship, patients’ attitude towards treatment, and social stigma [3, 69].

Electroconvulsive therapy has not been studied in the specific population of elderly patients with cancer. Therefore even if it is generally considered safe, it should be taken into consideration with caution and only in the case of patients who are refractory or intolerant to antidepressant drugs, suicidal or psychotic [110] (Table **2**).

## 4 DISCUSSION

Depression in older adults with cancer is a multiply-determined, heterogeneous disorder. Despite the high prevalence and deleterious effects of depression, older adults are far less likely to be accurately diagnosed or treated for depression compared to other age groups [4].

The principal finding of this systematic analysis are the following:

- The risk factors associated with depression in elderly people with cancer are numerous and can be divided into 4 main groups: 1) tumor-related, such as site of primary tumors (pancreatic, head and neck, lung and gynecological malignancies), stage of disease, high severity of cancer-related symptoms (severe and uncontrollable pain); 2) anticancer treatment-related, such as side effects of anticancer treatment, use of corticosteroids, specific chemotherapeutic drugs and endocrine therapy like tamoxifen; 3) patients-related: age (>80 years), personal history of previous depression and depression treatment, family history of depression and suicide, female gender, social isolation, the loss of a spouse, friend or relative, low educational levels, dependency, nutritional status, poor physical status, and increased physical impairment or discomfort; 4) the number and type of comorbidity: cognitive disorders and vascular risk factors, neuro-immune-endocrine system dysregulation (sympathetic/parasympathetic deregulation, thyroid function dysregulation).

- The overlap of the symptoms of cancer and the side effects of treatment with the symptoms of depression represents one of the main obstacles in diagnosing depression in elderly patients with cancer. Other important difficulties in diagnosing depression in this special group of patients derive from the different ways of reporting depressive symptoms of elderly people compared to younger adults, the different clinical types of depression with a moderate rate of major depressive disorder (MDD), and the high rate of minor depressive disorder accompanied by subthreshold forms of depression, the presence of somatic symptoms.

- The lack of data regarding which scale is the most appropriate to assess depression in geriatric patients with cancer.

- Patients with any mental illness, specifically co-occurring anxiety and depression, have an increased risk of diagnosis delay, of receiving non-definitive anticancer treatment, and of adjuvant chemotherapy delay compared to patients without a mental illness.

- Regarding suicidal risk, cancer and the diagnosis of mental disorders prior to cancer diagnosis correlate with an increased risk for suicide.

- Non-pharmacological therapeutic approach, pharmacological treatment and/or a combination of both can be used to treat elderly patients with cancer. In the choice of the most appropriate agent for elderly patients with cancer, previous response to an antidepressant in the past, patients’ global state of health and cognitive abilities, social and financial conditions, and other psychiatric disorders (anxiety, substance abuse, psychosis), must be considered The analysis of comorbidities and the assessment of polypharmacy are crucial in order to avoid potential side-effects and interactions between antidepressants and the other drugs taken by the patients.

## CONCLUSION

In our view, a possible strategy to improve the diagnosis of depression in elderly patients with cancer could be to seek the characteristic risk factors for depression of this group of patients early and act quickly on those that can be changed. Among these symptoms, pain plays an important role as it correlates with a greater risk of depression, especially when it is severe and not controlled by analgesic therapy. Doctors, especially the oncologist and the general practitioner, must pay more attention to this symptom which is often underestimated.

It is also necessary to establish specific diagnostic criteria for depression tailored to elderly patients with cancer and develop standardized tools for the age of patients that are able to separate somatic from affective symptoms of depression. In the absence of validated scales for assessing depression in elderly cancer patients, the use of multidimensional geriatric assessment in clinical practice could contribute to achieving this goal.

Finally, in elderly cancer patients, a detailed analysis of comorbidities should be made, especially those for which a role in the pathogenesis of depression is hypothesized (for instance, the dysregulation of thyroid function). Early treatment of these frequent comorbidities would contribute to improving the clinical management of depression in these patients.

Depressive symptoms in elderly patients with cancer should be followed throughout the course of the neoplastic disease because depression can also appear during chemotherapy and interfere with treatments’ adherence. The role of chemotherapy and treatment failure or success on depressive symptoms requires additional focused studies.

In conclusion, future research in elderly cancer patients should be conducted in order to provide data to delineate the most suitable modalities for the diagnosis and management of depression in this growing subset of cancer patients.

## Figures and Tables

**Fig. (1):**
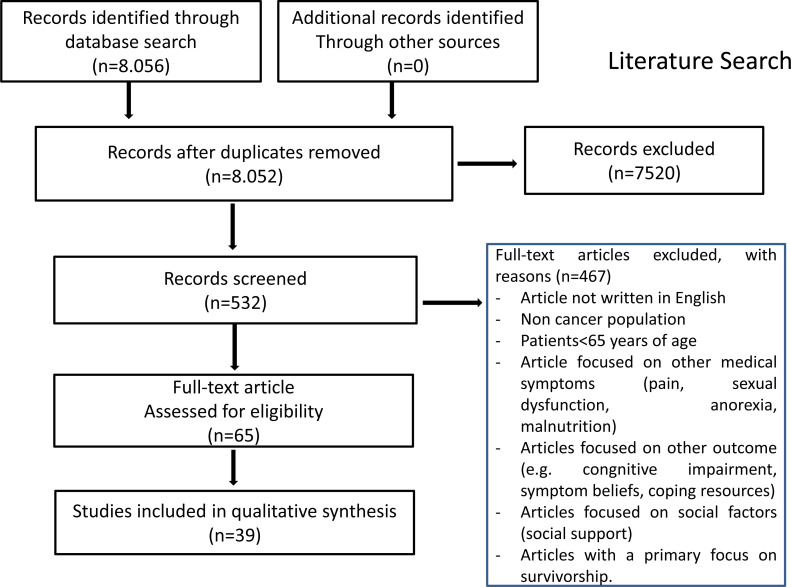
PRISMA flow diagram.

**Table 1:** 

**Depression DSM-5 Diagnostic Criteria** Five (or more) of the following have been present during the same 2-week period and represent a change from previous functioning; at least one of the same symptoms should be either (1) depressed mood or (2) loss of interest or pleasure. • Depressed mood most of the day, nearly every day, as indicated by either subjective report (*e.g*., feels sad, empty, hopeless) or observation made by others (*e.g*. appears tearful). (Note: in children and adolescents, can be irritable mood). • Markedly diminished interest or pleasure in all, or almost all, activities most of the day, nearly every day (as indicated by either subjective account or observation). • Significant weight loss when not dieting or weight gain (*e.g*. a change of more than 5% of body weight in a month), or decrease or increase in appetite nearly every day (note: in children, consider failure to make expected weight gain). • Insomnia or hypersomnia nearly every day. • Psychomotor agitation or retardation nearly every day (observable by others, not merely subjective feelings of restlessness or being slowed down). • Fatigue or loss of energy nearly every day. • Feelings of worthlessness or excessive or inappropriate guilt (which may be delusional) nearly every day (not merely self-reproach or guilt about being sick). • Diminished ability to think or concentrate, or indecisiveness, nearly every day (either by subjective account or as observed by others). • Recurrent thoughts of death (not just fear of dying), recurrent suicidal ideation without a specific plan, or a suicide attempt or a specific plan for committing suicide.

**Table 2:** Depression treatment strategies in elderly cancer patients.

**TREATMENT STRATEGY**	**OPTIONS**
Non-pharmacological: Psychotherapy	• Supportive Psychotherapy • Cognitive Behaviour Therapy (CBT) • Problem-Solving Therapy (PST) • Group Therapy
Pharmacological: Antidepressant drugs	• First Choice: Citalopram, Fluoxetine, Paroxetine, Sertraline (Serotonin Reuptake Inhibitors) • Second Choice: Venlafaxine, Mirtazapine, Bupropion • Third Choice: Nortryptiline, Desipramine
